# Of tuberculosis and non-tuberculous mycobacterial infections – a comparative analysis of epidemiology, diagnosis and treatment

**DOI:** 10.1186/s12929-020-00667-6

**Published:** 2020-06-17

**Authors:** Radha Gopalaswamy, Sivakumar Shanmugam, Rajesh Mondal, Selvakumar Subbian

**Affiliations:** 1 Department of Bacteriology, National Institute for Research in Tuberculosis, Chennai, India; 2 Public Health Research Institute, New Jersey Medical School, Rutgers, The State University of New Jersey, Newark, NJ United States

**Keywords:** Mycobacterium tuberculosis, Non-tuberculous mycobacteria, Lung disease, Molecular diagnosis, Drug sensitivity test, Antitubercular drugs, Macrolides

## Abstract

Pulmonary diseases due to mycobacteria cause significant morbidity and mortality to human health. In addition to tuberculosis (TB), caused by *Mycobacterium tuberculosis* (Mtb), recent epidemiological studies have shown the emergence of non-tuberculous mycobacteria (NTM) species in causing lung diseases in humans. Although more than 170 NTM species are present in various environmental niches, only a handful, primarily *Mycobacterium avium* complex and *M. abscessus*, have been implicated in pulmonary disease. While TB is transmitted through inhalation of aerosol droplets containing Mtb, generated by patients with symptomatic disease, NTM disease is mostly disseminated through aerosols originated from the environment. However, following inhalation, both Mtb and NTM are phagocytosed by alveolar macrophages in the lungs. Subsequently, various immune cells are recruited from the circulation to the site of infection, which leads to granuloma formation. Although the pathophysiology of TB and NTM diseases share several fundamental cellular and molecular events, the host-susceptibility to Mtb and NTM infections are different. Striking differences also exist in the disease presentation between TB and NTM cases. While NTM disease is primarily associated with bronchiectasis, this condition is rarely a predisposing factor for TB. Similarly, in Human Immunodeficiency Virus (HIV)-infected individuals, NTM disease presents as disseminated, extrapulmonary form rather than as a miliary, pulmonary disease, which is seen in Mtb infection. The diagnostic modalities for TB, including molecular diagnosis and drug-susceptibility testing (DST), are more advanced and possess a higher rate of sensitivity and specificity, compared to the tools available for NTM infections. In general, drug-sensitive TB is effectively treated with a standard multi-drug regimen containing well-defined first- and second-line antibiotics. However, the treatment of drug-resistant TB requires the additional, newer class of antibiotics in combination with or without the first and second-line drugs. In contrast, the NTM species display significant heterogeneity in their susceptibility to standard anti-TB drugs. Thus, the treatment for NTM diseases usually involves the use of macrolides and injectable aminoglycosides. Although well-established international guidelines are available, treatment of NTM disease is mostly empirical and not entirely successful. In general, the treatment duration is much longer for NTM diseases, compared to TB, and resection surgery of affected organ(s) is part of treatment for patients with NTM diseases that do not respond to the antibiotics treatment. Here, we discuss the epidemiology, diagnosis, and treatment modalities available for TB and NTM diseases of humans.

## Background

Tuberculosis (TB), caused by *Mycobacterium tuberculosis* (Mtb), is a leading killer among the infectious disease of humans that mainly affects the lungs [1]. Pathologically, TB is characterized as necrotizing granulomatous inflammation of infected organs [2]. Dissemination of Mtb infection in the population occurs mainly by inhalation of contaminated aerosols from patients with active pulmonary disease. In humans, exposure to Mtb can lead to either primary active disease or asymptomatic latent infection (LTBI) [3]. The LTBI accounts for about a third- to -quarter of the global population. These individuals are capable of reactivating to symptomatic TB upon host immune-suppressing conditions. The risk for LTBI cases to develop active TB is about 5% in the first 18 months of infection, after which the relapse rate reduces to nearly 5% for the lifetime [4].

The family of non-tuberculous mycobacteria (NTM) consists of about 170 species of mycobacteria. However, pulmonary diseases in humans are mostly caused by species of *M. avium* complex (MAC), *M. kansasii,* and *M. abscessus* [5]. Human infections due to NTM are primarily acquired from the environment, although the precise mode of transmission remains unclear. In addition to pulmonary involvement, lymphatic, skin, and soft tissues are also frequently affected by NTM infections [6]. Further, underlying health conditions, such as chronic obstructive pulmonary disease (COPD), pneumoconiosis, bronchiectasis, previous history of TB, post-radiotherapy fibrosis, chronic pulmonary aspiration, cystic fibrosis (CF), immune deficiency, HIV infection, alcoholism, cancer, and diabetes mellitus (DM) pose a significant risk for NTM infections [7].

In clinical specimens, differential diagnosis of Mtb and NTM species is a significant challenge and often misleading since both Mtb and NTMs show positivity to the conventional smear acid-fast staining method. Thus, the incidence of NTM has been underestimated in many TB-endemic countries.

The standard antibiotic regimen for the treatment of drug-sensitive TB contains isoniazid (INH), rifampicin (RIF), pyrazinamide (PZA), and ethambutol (ETH), administered for a minimum of 6 months. However, treatment of multi- and extremely-drug resistant (MDR and XDR) TB cases need additional antibiotics for a prolonged duration. With the availability of newer classes of drugs, such as bedaquiline and delamanid, novel regimens with shorter treatment periods are available to treat MDR-TB cases [8, 9]. In contrast, NTM diseases do not respond to anti-TB drugs [10]. Treatment of NTM diseases follows specific guidelines, based on the nature of infecting bacteria, and requires species identification. Unlike TB, the treatment for NTM disease takes at least 18 months, with 12 months sputum-negative period [6].

In both TB and NTM pulmonary diseases, the bacterial characteristics and the host factors influence the susceptibility and manifestations of infection as well as the outcome of treatment [11, 12]. Our understanding of the epidemiology, risk factors, and pathophysiology of pulmonary TB in humans has significantly improved over the past 50 years. However, these areas are underdeveloped for NTM diseases. Similarly, more diagnostic and treatment options are available for TB management, compared to NTM diseases. Nonetheless, promising new diagnostic methods and treatment modalities for all forms of TB and NTM disease are in the development pipeline. In this review, we evaluate the progress made in the areas of Mtb and NTM infections of humans, assessing mainly on the epidemiology, diagnosis, and treatment (Table 1).

**Table 1:** Summary of key features of pulmonary TB and NTM diseases

Category	Tuberculosis (TB)	Pulmonary Non-tuberculous mycobacteria (NTM) infections	References
1. Causative agent	*M. tuberculosis* complex organisms	*M. avium* complex. *M. abscessus, M. kansasii, M. malmoense*, *M. xenopi*	[2, 5, 6]
2. Mode of transmission	Inhalation of contaminated aerosols from patients with pulmonary TB	Primarily acquired from the environment - lack of person-to-person transmission	[3, 6]
3. Sex with higher disease burden	Male	Female	[1, 13]
4. Predisposition/Co-morbidities	HIV, DM, Immunosuppression	Bronchiectasis, Previous history of TB, CF, COPD	[1, 14–21]
5. Diagnosis			
5.1 Clinical	Lung involvement, alveolar infiltration, cavitation, lymphadenopathy and pleural effusion	Nodular or cavitary opacities on chest radiograph, or an HRCT scan that shows multifocal bronchiectasis with multiple small nodules	[6, 22]
5.2 Radiological	X-ray; rarely CT	X-ray; PET/CT or HRCT	[6, 22]
*Samples for the diagnostic test*	Sputum samples, bronchial or bronchioalveolar lavage aspirates, or tracheal aspirates; Gastric aspirate	Sputum, bronchial wash, or lavage; Gastric aspirate not preferred due to failure to indicate active pNTM disease	[22, 23]
5.3 Microbiological test			
*5.3.1 Staining*	Acid fast staining; Ziehl Neelson staining	Acid fast staining; Ziehl Neelson staining	[22–24]
*5.3.2 Culture*	Solid, Liquid - MGIT 960 system; the VersaTREK system; MB/BacT Alert 3D	Solid or liquid including MGIT but with PNB	[23, 25, 26]
5.4 Molecular biological test	TB Ag MPT64 RAPID **Nucleic acid amplification test (NAAT)** – Amplified Mycobacterium tuberculosis direct (MTD); Amplicor Mycobacterium tuberculosis Test; Xpert MTB-Rif system; Xpert MTB-Rif Ultra Loop-mediated isothermal amplification (LAMP)-based MTB detection system; Cross-priming amplification (CPA)-based TB diagnostic system; CE-IVD Genedrive; Anyplex II MTB/MDR and MTB/XDR; Anyplex MTB/NTM MDR-TB kit; EZplex MTBC/NTM; VereMTB Detection Kit **Line probe assays (LiPA)** – Inno-LiPA Mycobacteria assay; Genotype Mycobacterium CM and AS assays **Others -** MALDI-TOF Next gen sequencing **For Latent TB –** Tuberculin skin test IGRA test - QuantiFERON-TB Gold, QFT-GIT, QFT-Plus and T-SPOT.TB	TB Ag MPT64 RAPID – to differentiate M. tb complex from NTM HPLC PCR-RFLP ; PCR sequencing **NAAT**s – Accuprobe analysis Anyplex MTB/NTM MDR-TB EZplex MTBC/NTM; Genedia MTB/NTM Detection Kit **LiPAs** – Inno-LiPA Mycobacteria assay; GenoType Mycobacterium CM -AS **Others -** MALDI-TOF Next gen sequencing	**MPT64** - [27, 28] **HPLC** - [29, 30] **PCR-RFLP/Sequencing** - [31–34] **NAAT** – [35–53] **LIPA –** [49, 54–58] **Others –** [59–66] **LTBI –** [67–70]

## Main Text

### Epidemiology and transmission of TB and NTM

#### Epidemiology of drug-sensitive and drug-resistant TB

In 2018, about 1·5 million people died from TB, and nearly 10 million people fall ill with Mtb infection worldwide, of which only 6·4 million were diagnosed and officially reported. The extent of TB burden is higher in males (57%) than in females (32%). Globally, an estimated 1.7 billion people are latently infected with Mtb (LTBI) without obvious disease symptoms. Individuals with LTBI mostly develop active disease in the first 12 to 18 months, although reactivation can occur even decades after initial infection [1].

The incidence of global MDR/rifampicin-resistant (RR)-TB in 2018 was estimated to be 3.4% for new cases and 18% among previously treated cases, while the proportion of XDR-TB cases among MDR-TB cases was estimated at 6.2% [1]. Most of the drug-sensitive, MDR, and XDR cases are reported in Asian countries, including India and China. Inappropriate clinical use of anti-TB drugs and poor patient compliance, associated with prolonged multi-drug treatment, contributes to the emergence of drug resistant-Mtb strains [71]. Also, molecular epidemiological data suggest that transmission of MDR- and XDR- Mtb strains in the community is the dominant mode of spread in many TB-endemic countries [14].

Thus, a clear understanding of Mtb transmission and acquisition of new infection is essential for guiding effective TB control strategies [72]. Similar to drug-sensitive TB cases, there is a dire necessity to improve the diagnosis and treatment strategies for MDR- and XDR-TB cases, which is one of the main goals of the END-TB policy developed by the WHO [73].

#### Epidemiology of NTM diseases

The human infections due to NTM were earlier believed to be acquired mainly from contaminated environmental sources through aerosols; however, recent reports indicate person-to-person transmission as well [74, 75]. Several clinical reports of NTM cases revealed similarities in disease symptoms caused by NTM and TB [76, 77]. A study on population genomics shows that genetically clustered NTM organisms caused the majority of infections worldwide. This study has also revealed the recent emergence of dominant clones of *M. abscessus* that have spread between continents [74]. The incidence and prevalence of NTM cases and the strain distribution are highly variable across different geographical locations. A global survey of NTM species isolated from human specimens found that about one-half of them belongs to the *M. avium* complex (MAC). However, the relative frequency of MAC varies widely by geographical region - 31% of isolates from South America, 52% from North America, and 71% from Australia [78]. In a clinical study conducted among CF patients with NTM infection, MAC was isolated in 61%, *M. abscessus* in 39%, and other NTM in 21% of cases in at least one specimen. About 19% of these patients had multiple NTM species isolated [79]. Despite the heterogeneous distribution of NTM species worldwide, causing a spectrum of diseases, pulmonary NTM infections constitute a substantial, often unappreciated, burden of illness in humans [80]. Further, pulmonary NTM infections can occur without any co-existing chronic diseases, such as CF. A report by Marras *et al*. shows that the prevalence rate of NTM cases has increased in North America from 9.1 to 14.1 per 100,000 persons/year between 1997 and 2003 [81]. Interestingly previous studies had observed a reduction in the number of pulmonary NTM and TB cases in several countries after the implementation of national BCG vaccination policy. This suggests that BCG confers cross-protection against NTM, and in countries without a nationwide BCG immunization program, a rise in NTM cases is expected [82–85].

Importantly, unlike TB, pulmonary NTM infections are more prevalent in women (59%) and the elderly (median age 66) than younger men, with MAC being the most common NTM species [13]. Prevots *et al*., reported an increase in the prevalence of pulmonary NTM cases among individuals over 60 years, from 19.6 cases/100,000 person-years between 1994 and 1996 to 26.7 cases/100,000 person-years between 2004 and 2006. In this study, MAC was the most common species isolated in patients with definitive disease (80.1%) followed by *M. chelonae* and *M. abscessus* (12.1%), *M. fortuitum* (5.6%), and *M. kansasii* (5.5%) [86]. Similarly, an epidemiological study on the prevalence of pulmonary NTM diseases in Australia has found an increase in pulmonary NTM cases from 5.5 to 10.2/100,000 people over the six years (1999 to 2005), with the highest number of cases among people aged >60 years and predominantly women [87]. Further, the prevalence of pulmonary NTM diseases increased from 1.3 to 7.9 cases/100,000 population in Asia, most of which were due to MAC and *M. abscessus* [87, 88]. Moreover, in Europe, the prevalence of NTM cases has increased from 0.9 to 2.9/100,000 persons from 1995 to 2006, respectively [89]. Together, these studies indicate a growing trend in the incidence and prevalence of pulmonary NTM cases worldwide, in association with a range of underlying health conditions, such as immunosuppression, age, sex, and previous history of lung diseases.

#### TB among HIV and Diabetes Mellitus cases

Among HIV-infected individuals, TB is one of the most frequent opportunistic infectious diseases. The risk of developing TB is 26 times higher in people with HIV infection, compared to the non-HIV population [20]. This increased risk is due to the weakening of the immune system by HIV infection. The risk of developing TB is about 7% to 10% each year among HIV-positive individuals. In contrast, the risk of developing TB is 10% over a lifetime for people without HIV infection [14]. An estimated 8.6% (range, 7.4–10%) of the incident TB cases in 2018 were among people living with HIV infection [1].

Type 2 diabetes mellitus (DM) is another serious risk factor and critical co-morbid condition that significantly elevates the mortality due to TB worldwide [17]. Besides, the onset of DM aggravates the disease severity of TB cases and muddles with the response to treatment. Further, DM patients have a faster disease progression following Mtb infection, and they respond poorly to treatment [90–92]. Despite the increasing evidence on the profound impact of DM co-morbidity on TB treatment outcomes, data on TB-DM co-existence is missing in new TB drug clinical trials. Therefore, the inclusion of TB-DM co-morbidity should receive a higher priority in prospective randomized clinical TB drug efficacy trials, with specific emphasis on differential outcomes of treatment among TB-DM patients [18].

#### NTM diseases in HIV and Diabetes Mellitus cases

NTM infections do not present with the same clinical symptoms in patients with HIV/AIDS compared to “stand-alone” infections in otherwise healthy individuals. Besides, the possibility of overlooking NTMs in these patients is significant, since TB would be the first consideration among HIV-positive cases that show symptoms of pulmonary mycobacterial disease [15]. Both primary and secondary NTM infections can affect the respiratory system in humans. Unlike TB, a localized NTM pulmonary disease is rare in patients with HIV co-infection; instead, these cases typically display a disseminated form of the disease [19].

Although DM confers susceptibility to TB, the association between NTM infections and DM remains unclear. Few studies have indicated DM as a co-morbid condition in soft tissue and pulmonary NTM infections during baseline studies [16, 21, 93]. Thus, our understanding of the impact of DM on host immunity and disease progression during NTM infections needs to be significantly improved, and this should be an active area of future research.

### Diagnosis of TB and NTM diseases

#### Microbiological Diagnosis of TB

In general, diagnosis of pulmonary TB is suspected in patients with relevant clinical manifestations, such as persistent and productive cough, hemoptysis, fever, weight loss, and previous history of TB. The clinical observations of pulmonary TB can also be confirmed by chest X-ray findings, which is a routine practice in many TB-endemic countries [94]. In the X-ray of a patient with active pulmonary TB, alveolar infiltration, cavitation, lymphadenopathy, and pleural effusion are usually observed [22]. For pulmonary TB, the most prevalent form of the disease, the primary specimens are sputum samples, bronchial or bronchioalveolar lavage aspirates, or tracheal aspirates [68]. In TB-endemic countries, “Acid-fast bacilli (AFB) staining” or Ziehl–Neelsen staining procedure is the standard method of Mtb detection in sputum specimens [24]. The AFB staining has a sensitivity of up to 70% in the sputum specimen of patients with cavitary TB [68]. Thus, the AFB smear microscopy, although rapid and inexpensive, has limited sensitivity due to false positives, such as the presence of NTM in the samples [95]. Culturing the bacteria in liquid or solid media is the “gold” reference standard for TB diagnosis, since this method performs better than AFB staining, and is cost-effective in resource-poor countries [68]. While the AFB smear microscopy takes 12- 24 hours, the culture methods need 2--6 weeks to produce diagnostic results for TB.

There are three FDA-approved commercial diagnostic platforms available for the semi-automated, broth-based culture of mycobacteria: the Mycobacteria Growth Indicator Tube (MGIT) 960 system, the VersaTREK system, and the MB/BacT Alert 3D. These are an improvised version of conventional culture methods to detect Mtb; these methodologies take an average of 10 days for stable bacterial growth [25]. With these methods, the overall sensitivity of culture-confirmed TB cases can be increased from 91% with one sputum specimen to 98% and 100% with second and third sputum specimens, respectively. In TB-endemic countries, both solid and liquid culture methods are recommended with or without adjunct AFB smear microscopy [96, 97].

#### Microbiological Diagnosis of NTM diseases

In contrast to TB, diagnosis of NTM is very challenging due to exposure of individuals to the environment, and it is necessary to differentiate NTM isolate from an individual, as a commensal/colonizer, versus a causal agent of the disease. In fact, colonization of NTM species has been reported in many suspected NTM cases without any pulmonary disease symptoms. This observation highlights the ambiguity about whether a low-grade NTM infection or disease exists or if the specimen is contaminated with environmental NTM species [98]. The ATS (American Thoracic Society) and IDSA (Infectious Diseases Society of America) has issued a set of criteria to identify the real pulmonary NTM disease. This classification includes clinical findings, such as lung involvement on chest radiograph or PET/CT scan, microbiological testing, and strain identification, to confirm NTM disease [6].

Compared to TB, the clinical presentation of NTM is heterogeneous and complicated by underlying co-morbid conditions, such as DM, COPD, and CF [98]. Clinical symptoms, in combination with the presence of pulmonary nodular or cavitary opacities on the radiograph, or multifocal bronchiectasis, and dispersed multiple small nodules on HRCT scan, are indicative of active NTM disease [6]. Although AFB staining would identify mycobacteria, it would not discriminate NTM from Mtb. Therefore, it is recommended to grow NTM from the clinical specimens, such as sputum, bronchial wash, or lavage on a solid and/or liquid media. Culturing of mycobacteria on growth media is preferred for identifying rapid and slow-growing species and considered as a “gold standard” diagnostic method [23, 99, 100]

Identification of specific NTM species in the clinical specimen is crucial since the treatment regimens differ strikingly among different NTM strains. Various biochemical tests, including niacin accumulation test, arylsulfatase test, nitrate reduction, catalase estimation, and growth in MacConkey agar media, are commonly used for NTM species identification [101]. One of the vital biochemical tests used routinely in clinical laboratories to discriminate Mtb from NTM is the p-nitro benzoic acid test (PNB). In the PNB inhibition test, while the growth of Mtb is inhibited, NTMs grow on culture medium containing PNB, as they are resistant to PNB. The average reporting time for MGIT 960-PNB test is seven days, compared to 28 days for the LJ-PNB test and about one to two months when using the conventional biochemical analysis described above [26].

#### Molecular biological diagnosis of active TB

Since culture- and microscopy-based diagnostic techniques take a longer time to get results, molecular biological methods have emerged as a rapid diagnostic platform for TB. Introduction of the mycobacterial nucleic acid amplification test (NAAT) significantly reduced the turn-around time of clinical diagnosis of TB, compared to the traditional culture and smear methods [102]. The US-CDC and the Association of Public Health Laboratories (APHL) recommend the use of NAAT to diagnose TB in clinical specimens. Several commercial NAAT systems, such as the Amplified Mycobacterium tuberculosis Direct (MTD) test, Xpert MTB-RIFsystem, Amplicor Mycobacterium tuberculosis Test, loop-mediated isothermal amplification (LAMP)-based Mtb detection system, cross-priming amplification (CPA)-based TB diagnostic system and the CE-IVD Genedrive are widely used in clinical laboratories [64]. The MTD method, which is based on the Transcription-Mediated Amplification (TMA) and the Hybridization Protection Assay (HPA), qualitatively detects ribosomal ribonucleic acid (rRNA) from Mtb complex organisms within 2.5 to 3.5 hours [38]. Although the MTD test is helpful in rapid and reliable detection of Mtb, this test cannot ascertain drug susceptibility of the Mtb strain in the specimen [103]. The Amplicor Mtb Test is another PCR-based diagnostic tool for the direct detection of Mtb complex organisms [35]. Both MTD and Amplicor are Food and Drug Administration (FDA) approved NAAT systems for testing smear-positive TB cases [64]. In contrast, the Xpert MTB/RIF system is specific to TB diagnosis, designed for the GeneXpert platform, to detect drug-sensitive as well as RR- Mtb strains directly from the sputum sample. This assay is based on a nested real-time PCR amplifying the *rpoB* gene of Mtb, which is the most prominent target for rifampicin resistance, with high sensitivity (> 90%), and results can be obtained within 2 hours [41, 50]. The Xpert MTB/RIF Ultra was developed to overcome the drawback of identifying Mtb in paucibacillary TB cases, and is recommended by the WHO [51, 53]. The LAMP-MTBC detection kit, which is endorsed by the WHO, targets the *gyrB* gene and IS regions of the Mtb complex genome. In this assay, the amplified product is visualized with the naked eye or under ultraviolet (UV) light [47]. In the CPA-based TB diagnostic system, PCR amplification is done at a single temperature using multiple primers and probes of Mtb. The amplified products are detected on a lateral flow strip placed in an enclosed, sealed plastic device [39, 52]. The CE-IVD is a rapid molecular TB detection test designed with paper-based DNA extraction method, coupled with PCR amplification to detect a short repetitive region, rep13E12, and a segment of the *rpoB* gene of Mtb. Detection of reaction products requires the Epistem's Genedrive instrument, a portable, bench-top platform with real-time PCR and melting temperature analysis capabilities. However, this assay showed low sensitivity to MAC and cross-reacts with three other mycobacterial species, including *M. abscessus*, *M. gordonae*, and *M. thermoresistibile* [36, 46].

Apart from Xpert MTB.-RIF, newer kits are available to diagnose MDR- and XDR- TB cases. The Anyplex II MTB/MDR and MTB/XDR are designed to detect resistance mutations in the Mtb genome, including *rpoB* (RIF), *katG,* and *inhA* (INH), *gyrA* (fluoroquinolones), and *rrs* and *eis* (aminoglycosides) [40, 44]. Further, the Anyplex MTB/NTM MDR-TB kit has also been evaluated to detect Mtb complex and NTM species, as well as their drug susceptibility to RIF (*rpoB*) and INH (*katG* and *inhA*) [45]. The EZplex MTBC/NTM is a Real-Time PCR kit used to detect Mtb complex and NTMs. This assay has an overall sensitivity and specificity of 98.6%-100% for the detection of Mtb [42]. The VereMTB Detection Kit is a NAAT based Lab-on-Chip (LoC) assay used for the detection and identification of Mtb complex, NTMs, and the diagnosis of MDR-TB. This kit targets *IS6110* and *16srRNA* sequences to differentiate Mtb from NTM. Further, the LoC kit can be used on direct sputum samples to perform DST, to identify resistance mutations in *rpoB* (RIF) and *katG/inhA* (INH). This kit has a sensitivity and specificity of 97.0% and 98.3% for the detection of the Mtb complex [43].

The Line probe assays (LiPA) are another molecular technique that makes use of hybridization-based probes for the identification of mycobacteria from samples [58]. The LiPA utilizes nitrocellulose membrane strips embedded with genus- and species-specific probes. The turn-around time of the LiPA method is approximately six hours, including a preliminary PCR amplification [58]. There are three commercial LiPA kits available – the Inno-LiPA Mycobacteria assay, the Genotype Mycobacterium CM, and AS assays. These assays target the 16S-23S rDNA spacer region and the 23S rDNA for the identification of mycobacteria [33]. The Inno-LiPA Mycobacteria is a reverse-hybridization, DNA probe assay platform, designed to identify up to 17 different taxa simultaneously, with a sensitivity of 99.6%. The Genotype MTBC is another commercially available DNA strip assay used to differentiate members of the Mtb complex organisms and the identification of *M. bovis* BCG. Further, this method can be performed directly on liquid cultures of mycobacteria from primary isolations without the need for further cultivation on solid media [55].

Whole genome sequencing (WGS) is an alternate option to identify and characterize various mycobacterial species; however, it incurs a higher cost and is not suitable for routine diagnostic screening of samples in endemic countries. There are first and second-generation (next-gen) sequencing (NGS) methods that help to detect mutations, polymorphisms that are responsible for drug resistance in Mtb [64]. Previously, the need for extraction of DNA from cultured samples discouraged the use of sequencing as a routine diagnostic test [65]. However, with the development of a system to use the patients’ sputum samples directly, NGS is evolving as a popular direct-diagnostic tool for TB diagnosis [59, 63, 65, 66].

The Matrix-Assisted Laser Desorption Ionization-Time Of Flight (MALDI-TOF) technology, in conjunction with mass spectrometry (MS), is another method for the identification and differentiation of mycobacteria. This method has 98.6% specificity and takes approximately 1–2 h to generate results from samples and is highly cost-effective [62].

#### Diagnosis of LTBI

Unlike the diagnosis of active TB, only a few methods are in use to diagnose LTBI cases. These are individuals with immunological evidence of exposure to Mtb or its antigens but without symptomatic clinical disease. Two commonly used screening tests for LTBI are the tuberculin skin test (TST) and the Interferon-gamma release assay (IGRA). The conventional TST involves an intradermal injection of purified protein derivative (PPD), followed by an examination of the induration after 48 – 72 hours [67]. Individuals previously exposed to Mtb and/or its antigens are sensitized to PPD and produce cytokines at the site of injection that causes a delayed-type hypersensitivity reaction. Conventionally, an induration of >10mm to PPD-injection, is considered a positive response in individuals without HIV or other co-existing health conditions. However, BCG vaccinated individuals can also show a hypersensitivity reaction, making the TST as a non-specific screening test [67].

The IGRA is used for LTBI screening of BCG vaccinated individuals and those of > 5 years of age, with a low or moderate risk of developing the active disease [68]. Currently, two types of IGRAs are available: QuantiFERON-TB Gold In-tube Test (QFT-GIT) and T-SPOT.TB. These assays use Mtb-specific antigens and measures the host immune cell response (i.e., production of interferon-gamma; IFNg); however, they differ technically and their target readout [67]. While the T-SPOT.TB is an ELISPOT assay that uses peripheral blood mononuclear cells (PBMCs) isolated from the whole blood; the QFT-GIT is an ELISA-based test for measuring IFNg secreted by PBMC upon Mtb antigen stimulation. The QFT-GIT tubes are precoated with a single cocktail of peptides from Mtb proteins, such as ESAT-6, CFP-10, and TB7.7 [69]. The QFT-GIT IGRA received US-FDA clearance in 2017. An improvised version of QFT-GIT, namely QFT-Plus-IGRA, provides a diagnostic advantage over the previous version, mainly to diagnose cases with CD8^+^ T-cell-reliant diseases, such as HIV-TB. However, this assay is not cost-effective and needs further evaluation to use in TB-endemic countries [70].

#### Drug susceptibility testing for TB

A critical feature of TB diagnosis is identifying the drug susceptibility of Mtb to the first and second-line antibiotics rapidly and accurately for efficient treatment. While MDR-TB cases are resistant to the first-line drugs, INH and RIF, XDR-TB cases are resistant to INH, RIF, any fluoroquinolones and, at least one of the three injectable second-line drugs (amikacin, kanamycin, and capreomycin) [104, 105]. The standardized drug susceptibility testing (DST) procedures require eight to 12 weeks to determine drug-resistant Mtb on solid growth media. The DST by automated liquid culture systems is rapid and has better sensitivity than growing bacterial culture on conventional liquid or solid media. However, even with liquid cultures, two to four weeks are needed to obtain DST for suspected MDR/XDR cases.

The MGIT system has replaced the conventional BACTEC 460 TB radiometric system of DST and is currently used in several TB-endemic countries [106]. This method reduced the turn-around time of DST to approximately ten days.

Apart from the liquid-based assays, rapid phenotypic methods, such as growth in tyrosine kinase medium, microscopic observation of drug susceptibility (MODS), and FASTPlaque-Response assays have also been evaluated in MGIT platform, since patient samples can directly be tested in this methods [107]. Despite their technical advancement, these procedures lack reliability and need another confirmatory test for concordant DST results. Recently, WGS is gaining more attention as an option to detect drug-resistant Mtb and for better understanding of the molecular basis of TB transmission in the population. In general, a good correlation exists between Mtb genetic mutations and culture-based DST results in the context of TB treatment [108]. Abbott RealTime MTB RIF/INH Resistance kit was evaluated and found effective in detecting RIF and INH resistance [109]. Several diagnostic kits described above are also evaluated for direct DST using sputum samples. For example, VereMTB kit showed a sensitivity and specificity of 85.7% and 93.9%, respectively, for RIF resistance detection and 75.0% and 95.7%, respectively, for INH resistance detection [43].

#### Molecular biological diagnosis of NTM diseases

Unlike TB, the methodologies used to diagnose NTM diseases, to discriminate between Mtb and NTM and, to differentiate among NTM species, are complicated and underdeveloped. Rapid differentiation between Mtb and NTM, as well as species identification in the clinical sample, is crucial for effective treatment. A quick Immunochromatographic test (ICT) kit, called SD Bioline TB Ag MPT64 RAPID®, has been routinely used for the differentiation of Mtb from NTM. The ICT kit detects MPT 64 antigen, present only in Mtb isolates, using mouse monoclonal antibody. This assay takes less than 30 minutes, does not require special instrumentation or sample preparation, and has been extensively evaluated for its reliable performance [27, 28]. Another method for NTM species identification is HPLC, in which mycolic acids, a peculiar type of cell wall fatty acids found in mycobacteria, are analyzed. The HPLC method is highly species-specific and has been shown to differentiate 99.5% of mycobacteria, cultured from clinical specimens, and 95.6% of NTM species [29, 30].

In addition, Plikaytis *et al* have developed a two-step assay by combining gene amplification and restriction fragment length polymorphism analysis for differentiating among slow-growing mycobacteria. This method can differentiate >90% of potentially pathogenic mycobacteria isolates and >86% of all strains in clinical specimens, including Mtb, *M. bovis*, *M. avium*, *M. intracellulare*, *M. kansasii*, and *M. gordonae* [32]. A further improvement in this technique showed PCR and PRA for almost 20 NTM strains with the recognizable pattern established for each of them [34].

A PCR-based sequencing technique has become the gold standard for the identification of mycobacterial species. In this method, the gene coding for 16S ribosomal RNA, containing both conserved and variable regions, and present in all bacterial species are most commonly targeted for amplification and sequencing [31, 33].

Molecular methods also help in NTM species identification in clinical samples. Accuprobe analysis, which involves nucleic acid hybridization assay that allows rapid identification of Mtb complex, MAC, *M. intracellulare*, *M. gordonae,* and *M. kansasii* is one of the most extensively used methods [37, 49].

The Anyplex MTB/NTM MDR-TB was also evaluated to differentiate NTMs from the Mtb complex [45]. The EZplex MTBC/NTM kit and VereMTB detection kit described earlier were also evaluated and shown to be useful for detecting NTM species directly from a sputum sample. The sensitivity and specificity of this method were 98.8%-100% for NTM [42, 43]. The Genedia MTB/NTM Detection Kit is a multiplex real-time PCR assay that targets the IS*6110* region of Mtb, and the internal transcribed spacer region and the *rpoB* gene of NTM. This kit has shown efficient differential detection of NTM in smear-positive cases, but inadequate for smear-negative cases, and hence needs further validation [48].

Also, an INNO-LiPA Mycobacteria assay, targeting the 16S-23S rRNA spacer region, has been developed for the detection of *Mycobacterium* spp. and identification of members of the Mtb complex and differentiating 15 NTM species, with an accuracy of 99.6% [57, 58]. Similarly, the GenoType Mycobacterium CM test is capable of identifying the Mtb complex and an additional 24 NTM species. In comparison, the GenoType Mycobacterium AS test is capable of identifying an additional 19 NTM species [54, 56].

As mentioned above, the MALDI-TOF/MS has also been used to identify NTM species from clinical specimens. This method is accurate, rapid, and appears cost-effective system; however, sophistication in instrumentation limits the use of this technique for routine use in endemic countries [60, 61].

#### Drug susceptibility testing for NTM diseases

After the identification of mycobacterial species present in the clinical specimen, it is crucial to determine their drug susceptibility pattern to devise a suitable treatment strategy. Compared to TB, the DST for NTM diseases is difficult and controversial because of inconsistent agreement in results observed between *in vitro* testing and *in vivo* clinical outcomes. Pulmonary NTM disease is a common manifestation among the affected individuals, and the most frequently encountered NTM species are subjected to DST by the broth dilution method [110]. Since a combination of antibiotics is used to treat NTM disease, checkerboard assays, or time-kill analysis are preferred to assess the *in vitro* drug synergy [111, 112]. The availability of DST results, along with diagnosis, is very crucial in developing an ideal treatment strategy for NTM diseases.

### Treatment of TB and NTM diseases

#### Treatment of drug-sensitive TB cases

For efficient management of TB, treatment should be initiated as early as possible in the confirmed as well as suspected cases. Delays in starting treatment have been closely associated with poor clinical outcomes [113, 114]. The currently used anti-TB drugs are classified into: group 1- first-line oral antibiotics, INH, RIF, ETH, and PZA; group 2- injectable second-line medicines (amikacin, kanamycin, and capreomycin plus streptomycin, which is considered first-line, but also an injectable); group 3- fluoroquinolones; group 4- second-line bacteriostatic drugs (ethionamide/prothionamide, para-aminosalicylic acid, and cycloserine/terizidone); and group 5- new or repurposed drugs [115]. The ATS, IDSA and the US-CDC have jointly developed a guideline for the treatment of drug-susceptible TB, which is endorsed by the US National Tuberculosis Controllers Association (NTCA) and the European Respiratory Society (ERS) (Table 2). According to this guideline, the preferred regimen for treating adults with TB caused by organisms that are not known or suspected to be drug-resistant should consist of INH, RIF, PZA, and EMB for two months, followed by four months of INH and RIF. In the case of drug susceptibility data available, EMB can be discontinued if the isolate is susceptible to both RIF and INH [116]. For MDR and XDR-TB cases, additional drugs from class-2 through 5 can be prescribed based on the clinical manifestation of disease and the drug-resistance pattern of the bacteria (see below).

**Table 2:** Description of drugs used in the treatment of adults with TB based on the ATS/ CDC/ IDSA Guidelines

Drug Group	Anti -TB drug	Dosage
First-line^a^	Isoniazid (Oral or IM or IV)	5 mg/kg (~ 300 mg) Pyridoxine 25–50 mg/day, is given with INH to persons at risk of neuropathy
	Rifampicin (Oral or IV)	10 mg/kg (~ 600 mg)
	Pyrazinamide (Oral)	1000 mg (40 - 55 kg weight) 1500 mg (56 – 75 kg weight) 2000 mg (76 – 90 kg weight)
	Ethambutol (Oral)	800 mg (40 - 55 kg weight) 1200 mg (56 – 75 kg weight) 1600 mg (76 – 90 kg weight)
Second-line^a^	Cycloserine (Oral)	10–15 mg/kg (usually 250–500 mg once or twice daily
	Ethionamide (Oral)	10–15 mg/kg (usually 250–500 mg once or twice daily
	Streptomycin (IM or IV)	15 mg/kg daily. Some clinicians prefer 25 mg/kg 3 times weekly or the 15 mg/kg dose 3 times weekly for patients with poor renal function
	Amikacin/kanamycin (IM or IV)	15 mg/kg daily. Some clinicians prefer 25 mg/kg 3 times weekly or the 15 mg/kg dose 3 times weekly for patients with poor renal function
	Capreomycin (IM or IV)	15 mg/kg daily. Some clinicians prefer 25 mg/kg 3 times weekly or the 15 mg/kg dose 3 times weekly for patients with poor renal function
	Para-amino salicylic acid (Granules or tablets or IV)	200–300 mg/kg total (usually divided 100 mg/kg given 2 to 3 times daily)
	Levofloxacin (Oral or IV)	500–1000 mg daily
	Moxifloxacin (Oral or IV)	400 mg daily
Anti-TB drugs with limited data available on safety and effectiveness ^b^	Bedaquiline (Oral)	400 mg daily for 14 days followed by 200 mg 3 times/wk
	Linezolid (Oral or IV)	600 mg daily
	Clofazimine (Oral)	100 mg daily
	Delamanid (Oral)	100 mg twice daily
	Meropenem (IV)	1,000 mg 3 times/day
	Imipenem–cilastatin (IV)	1,000 mg 3–4 times/day
	Clavulanate (Oral or IV)	250 mg 3 times/day
	High-dose isoniazid (Oral or IV)	15 mg/kg daily

Ref ([47]^a^[33]^b^). *IM* Intra-muscular; *IV* Intravenous. *ATS* American Thoracic Society; *CDC* Centres for Disease Control; *IDSA* Infectious Disease Society of America.

#### Treatment of HIV-TB, TB-DM, and drug-resistant TB cases

The treatment regimen for patients with HIV-TB have many important considerations, compared to TB cases without HIV. For drug-susceptible pulmonary TB cases with HIV infection, receiving anti-retroviral therapy (ART), the standard TB regimen with two months of INH, RIF, PZA, and EMB followed by four months of INH and RIF, is recommended [117]. In these patients, ART should be started within the first eight weeks of beginning the anti-TB treatment and within two weeks in profoundly immunosuppressed HIV-TB patients with a low CD4 cell count ((<50 cells/mm^3^) [1]. Further, the WHO recommends the use of cotrimoxazole prophylaxis for all HIV-infected people with active TB, regardless of the CD4 cell counts, to manage co-infections. However, early initiation of ART combined with anti-TB drug results in a condition known as immune reconstitution inflammatory syndrome (IRIS), marked by fever, enlarged lymph nodes, elevated pulmonary infiltrates, and exacerbated inflammatory changes in various internal organs. Usually, IRIS develops in patients within the first three months of ART and is more common when the CD4 cell counts are low (<50 cells/mm^3^). To manage patients with IRIS, anti-inflammatory agents, including corticosteroids, are added to the TB and ART therapy [118–120]. In patients with TB-DM, the administration of metformin has been shown to reduce the risk of TB, reduce the mortality during treatment, improve sputum-culture conversion rate, and reduce the relapse rate of TB [79, 121–123]. Additionally, metformin has been considered as a potential adjunctive host-directed therapeutic (HDT) agent to treat TB, but this concept warrants more rigorous clinical research to optimize along with TB regimen [124–126].

Although a whole battery of anti-TB drugs is available, the clinical outcome of treatment in different types of pulmonary and extrapulmonary TB is highly variable. Often the therapy achieves an incomplete cure, due to several reasons, including prolonged duration of treatment, drug-induced adverse side effects, patient non-compliance, and the existence of co-morbidities, such as HIV. As a complementary therapeutic modality to antibiotics, a new avenue has been developed, which focusses on host-directed therapy (HDT) for the treatment of TB. This novel concept is based on the modulation of the host response to Mtb infection with small molecules, and used mostly in conjunction with antibiotics, to achieve better and rapid control of TB. The small molecule HDT agents that target autophagy, vitamin D pathway, and anti-inflammatory response are tested in pre-clinical animal models, and several are in phase 2 clinical trials. The case of HDT for TB therapy has been extensively reviewed and published elsewhere recently by several researchers [124, 127, 128].

Treatment of patients with drug-resistant TB follows a different regimen, based on the resistance pattern of infecting Mtb. For MDR-TB, the WHO recommends a 9 – 12-month regimen containing seven drugs (gatifloxacin, ETH, PZA, INH, clofazimine, kanamycin, and prothionamide ) during the first four months, followed by four drugs (gatifloxacin, ETH, PZA, and INH) for the next five months [129]. A limitation of this “Bangladesh regimen” is the resistance of bacilli to one or more drugs, making it essential to have additional drug combinations to replace accordingly. With the availability of newer TB drugs, such as bedaquiline, delamanid, pretomanid, and other repurposed drugs, the oral treatment strategy for MDR-TB has been improved [130]. The clinical approach for MDR-TB management incorporates the evidence-based review of the performance of individual medicines used at the recommended doses, with consideration of the balance of benefits versus harms for each drug, the experience of MDR-TB experts on the committee, as well as the perspectives of patients (Table 2). The final choice of drugs and drug classes is also contingent on those considerations, in addition to appropriate monitoring of patients for significant adverse effects, drug-drug interactions, co-morbidities, and drug availability.

Similar to MDR-TB, XDR-TB cases are also treated with a combination of available drugs, based on the drug resistance pattern of infecting Mtb. Although conventional MDR-/XDR-TB treatment strategies include surgery as one of the first therapeutic approaches, this strategy has been changed in recent decades, with the invention of several new classes of antibiotics. Apart from the standard anti-TB drugs, there are other drug classes already in clinical use for TB (fluoroquinolones, diarylquinolines, riminophenazines, carbapenems, oxazolidinones, nitroimidazoles), which have been redesigned to optimize bioavailability, potency, safety, or activity against resistant Mtb strains [131]. Besides, new classes of drugs (e.g., inhibitors of Mtb DprE1, leucyl-tRNA synthetase, cholesterol catabolism) with novel mechanisms of action have also been evaluated for MDR/XDR-TB treatment. Several of these compounds are in phase 1 or phase 2 clinical trials and extensively reviewed recently [131]. However, several scientific societies, national and international medical organizations, suggest surgery as adjunctive therapy for MDR/XDR-TB [9].

#### Treatment of pulmonary NTM diseases

Unlike the Mtb complex family with few member species, pathogenic NTM is comprised of about 170 species. Each of these NTM species has been identified to have a role in various diseases, including pulmonary disease, lymphadenitis, disseminated diseases, skin, and soft tissue infections [80]. Pulmonary NTM disease is predominantly caused by five of the NTM species (MAC, *M. abscessus*, *M. kansasii, M. xenopi*, and *M. malmoense*), with a different incidence of each of the strains, worldwide [6].. In general, the treatment outcome of pulmonary NTM diseases is highly variable and determined by the host- and pathogen-derived factors, and the nature of the treatment regimen. Despite the use of multiple antibiotics, sputum-conversion, from a positive-to-negative finding of bacteria, is often difficult to achieve in NTM cases, especially those infected with macrolide-resistant NTM species. Resection surgery, in a subgroup of patients with a focal disease or persistent symptoms, has also been considered as adjuvant therapy for severe pulmonary NTM disease [132]. Although NTMs are implicated in many extrapulmonary disease conditions, in this review, we focus on the treatment of pulmonary diseases.

For most patients with nodular or bronchiectatic MAC lung disease, a three-times-weekly regimen containing clarithromycin or azithromycin, ETB, and RIF is recommended as initial therapy (Table 3) [6, 133]. This intermittent therapeutic approach has the potential advantages of lower medication costs and fewer drug-induced adverse side effects. However, for more severe cavitary disease due to MAC species, the addition of injectable amikacin or streptomycin, along with daily dosing of the above mentioned standard drugs, is preferred [6, 133]. The clarithromycin-resistant MAC cases with severe disease should be treated with RIF, ETB, and INH or a quinolone (e.g., moxifloxacin), and an injectable aminoglycoside (amikacin or streptomycin). Wherever required, the injectable aminoglycoside therapy is replaced with the nebulized amikacin. Importantly, the antibiotic treatment for MAC-pulmonary disease should be continued for a minimum of 12 months after culture conversion [6, 133]. The clofazimine inhalation suspension therapy has been tested in mouse models of *M. avium* and *M. abscessus* and proven to be promising, with the drug accumulating at four times more concentration than the oral dosing, in the lungs. This therapy is well tolerated and effective, and now a novel therapeutic candidate for further validation to use in NTM therapy [134].

**Table 3:** Description of drugs used in the treatment of adults with NTM diseases based on the ATS/IDSA/BTS Guidelines

NTM Species	ATS/IDSA Guidelines ^a^	BTS Guidelines ^b^
*M. abscessus*	Minimal surgical resurrection Macrolide (Clarithromycin 500–1,000 mg/day); Intravenous amikacin; streptomycin or cefotaxime	**Clarithromycin-sensitive or inducible macrolide-resistant isolates** ***Initial phase: ≥1 month*** intravenous Amikacin 15 mg/kg daily or 3×per week; intravenous Tigecycline 50 mg twice daily; where tolerated intravenous Imipenem 1 g twice daily; where tolerated oral Clarithromycin 500 mg twice daily or oral Azithromycin 250–500 mg daily. ***Continuation phase****:* nebulized Amikacin and oral Clarithromycin 500 mg twice daily or azithromycin 250–500 mg daily plus oral clofazimine 50–100 mg daily; oral linezolid 600 mg daily or twice daily; oral minocycline 100 mg twice daily; oral moxifloxacin 400 mg daily; oral cotrimoxazole 960 mg twice daily based on the guidance of DST and patient tolerance. **Constitutive macrolide-resistant isolates** Same as above, except Clarithromycin is omitted in both phases.
*MAC* *(M. avium complex)*	**Non-severe MAC-PD** Clarithromycin 1,000 mg or Azithromycin 500 mg, Ethambutol 25 mg/kg, and Rifampin 600 mg administered three times per week **Severe MAC-PD** Clarithromycin 500–1,000 mg/day; Azithromycin 250 mg/ day; Ethambutol 15 mg/kg/day; Rifampin 10 mg/kg/day all daily; Intravenous drugs like amikacin or streptomycin	**Non-severe MAC-PD** Rifampicin 600 mg 3 x per week; Ethambutol 25 mg/kg 3×per week; Azithromycin 500 mg 3×per week or Clarithromycin 1 g in two divided doses 3 x per week. **Severe MAC-PD** Rifampicin 600 mg daily; Ethambutol 15 mg/kg daily; Azithromycin 250 mg daily or Clarithromycin 500 mg twice daily; Intravenous amikacin for up to 3 months or nebulized amikacin **Clarithromycin-resistant MAC PD** Rifampicin 600 mg daily; Ethambutol 15 mg/kg daily; Isoniazid 300 mg (+pyridoxine 10 mg) daily or moxifloxacin 400 mg daily Intravenous amikacin for up to 3 months or nebulized amikacin
*M. kansasii*	**Rifampicin-sensitive*M. kansasii*-PD** Rifampin 10 mg/kg/day; Ethambutol 15 g/kg/day; Isoniazid 5 mg/kg/day; Pyridoxine (50 mg/day) - Daily **Rifampicin-resistant*M. kansasii*-PD** Three drug regimen – with guidance from DST	**Rifampicin-sensitive*M. kansasii*-PD** Rifampicin 600 mg daily; Ethambutol 15 mg/kg daily; Isoniazid 300 mg (with pyridoxine 10 mg) daily or azithromycin 250 mg daily or clarithromycin 500 mg twice daily. **Rifampicin-resistant*M. kansasii*-PD** Three drug regimen – with guidance from DST
*M. malmoense*	Isoniazid; Rifampicin; Ethambutol; With or without Quinolones and Macrolides with guidance from DST (dose not specified)	**Non-severe*M. malmoense-*PD** Rifampicin 600 mg daily; Ethambutol 15 mg/kg daily; Azithromycin 250 mg daily or Clarithromycin 500 mg twice daily. **Severe*M. malmoense-*PD** Same as above plus intravenous amikacin for up to 3 months or nebulized amikacin.
*M. xenopi*	Isoniazid; Rifabutin or Rifampin; Ethambutol, and Clarithromycin, with or without an initial course of Streptomycin plus inclusion of a Quinolone, preferably moxifloxacin to be substituted for one of the anti-tuberculous drugs	**Non-severe*M. xenopi*-PD** Rifampicin 600 mg daily; Ethambutol 15 mg/kg daily; Azithromycin 250 mg daily or Clarithromycin 500 mg twice daily; Moxifloxacin 400 mg daily or Isoniazid 300 mg (+pyridoxine 10 mg) daily **Severe*M. xenopi*-PD** Same as above plus intravenous amikacin for up to 3 months or nebulized amikacin.

Ref ([93]^a^[87]^b^). *PD* Pulmonary disease; *DST* Drug sensitivity testing, *ATS* American Thoracic Society; *IDSA* Infectious Disease Society of America; *BTS* British Thoracic Society.

In contrast to MAC, *M. abscessus* is resistant to all front-line anti-TB drugs, and hence medications of choice are oral macrolides, combined with two parenteral drugs. The ATS/IDSA guidelines recommend administration of amikacin, cefotaxime, or imipenem for 12 months or till sputum culture conversion [6]. However, *M. massiliense*, one of three subspecies of *M. abscessus,* shows a characteristically different response to clarithromycin, compared to *M. abscessus.* Therefore, precise diagnosis and identification of these species are crucial for the treatment of infected patients [135, 136]. The British Thoracic Society (BTS) guidelines recommend an intravenous injection of amikacin, tigecycline, and imipenem, in addition to an oral macrolide. However, for macrolide-resistant *M. abscessus*, only amikacin, tigecycline, and imipenem are used during the initiation phase (Table 3). Additionally, for both strain types, oral administration of nebulized amikacin, clarithromycin (only for macrolide sensitive type), followed by one or more drugs, such as clofazimine, linezolid, minocycline, moxifloxacin, cotrimoxazole is recommended, based on drug susceptibility of the strain and patient tolerance to the drugs (Table 3). Antibiotic treatment for pulmonary diseases caused by *M. abscessus* should be continued for a minimum of 12 months, after specimen culture conversion [133].

Diseases due to *M. kansasii* remains easily treatable since it has similar disease/pathology presentation as pulmonary TB, characterized by fibro-cavitary lesions in the upper lung lobes and usually susceptible to anti-TB drugs with frequent occurrence of RR strains [6]. The ATS/IDSA guidelines recommend a daily regimen of RIF, ETB, INH, and pyridoxine for drug-sensitive *M. kansasii*; for RR strains, a three-drug regimen, including clarithromycin or azithromycin, moxifloxacin, ethambutol, sulfamethoxazole, or streptomycin is recommended [6]. The BTS guidelines are slightly different from the ATS/IDSA counterpart and suggest to include a macrolide, such as azithromycin or clarithromycin, as an alternative to INH combined with pyridoxine. Treatment of drug-resistant *M. kansasii* strains requires the use of the same three antibiotic therapy, as suggested by ATS/IDSA guidelines [133]. Treatment duration for *M. kansasii* lung diseases should also include 12 months after negative sputum culture result (Table 3).

Most of the infections involving *M. malmoense* have been associated with pulmonary and lymph node diseases; however, the pulmonary form of the disease is more challenging to treat [6]. The ATS/IDSA guidelines recommend the use of INH, RIF, ETB with and without quinolones and macrolides [6]. The BTS guidelines recommend a minimum of three-drug regimen, including RIF, ETB, and macrolide as a daily treatment for less severe disease. For severe cases, the use of additional injectables, such as amikacin or streptomycin for three months is recommended; however, the injectable can be replaced with the nebulized form of amikacin (Table 3) [133].

Concerning *M. xenopi*, neither an optimal treatment regimen nor a stipulated duration of treatment has been established. Further, the response of *M. xenopi* to various antibiotics is variable and does not always correlate well with the results of *in vitro* DST results [6]. A reasonable regimen for *M. xenopi* infection should consist of INH, rifabutin or RIF, ETB, and clarithromycin, with or without an initial course of streptomycin and a quinolone, preferably moxifloxacin, to be substituted for one of the anti-tuberculous drugs. After the initiation of treatment, the sputum conversion occurs readily among *M. xenopi-* infected cases; however, the relapse rates are high even when the regimen contains a macrolide [6]. The BTS guidelines give a choice of four-drug regimen comprising of RIF, ETB, and a macrolide (clarithromycin or azithromycin), with either a quinolone (ciprofloxacin or moxifloxacin) or INH. For severe NTM diseases, injectable or nebulized amikacin is added to the above regimen for up to 3 months (Table 3). The BTS guidelines also suggest continuing treatment for up to 12 months after culture conversion [133].

#### Complications of NTM disease treatment

The treatment of NTM disease is more complicated and associated with a higher rate of toxicity-related issues compared to TB treatment. The standard, multi-drug therapy used for NTM lung diseases can cause significant adverse effects, which leads to treatment discontinuation and patient non-compliance. Therefore, drug-induced hepatotoxicity, due to rifampin, macrolides, imipenem, or tigecycline, should be carefully monitored, in addition to hematologic disturbances, as assessed by blood cell counts. Further, macrolides can cause ototoxicity or vestibular dysfunction [137]. For efficient management of NTM diseases, these medications should be reviewed periodically, and side effects should be carefully monitored throughout the treatment. The *in vitro* DST for many NTM does not correlate well with the clinical response of patients to anti-mycobacterial drugs. Additionally, the procedures for NTM drug susceptibility are limited, compared to TB [6].

Since a significant level of heterogeneity exists in patient response to treatment and that sputum conversion is not reliably achieved in many cases, the medical management of NTM diseases is more complicated than that of TB. Moreover, minimal resection surgery, in a carefully selected subgroup of patients with a focal illness or persistent symptoms, is considered as adjuvant therapy in the treatment of pulmonary NTM infection [132]. Importantly, several new drugs are in the pipeline to improve the treatment of NTM diseases, which include oxazolidinone (Linezolid, Tedizolid, LCB01-0371), inhaled nitric oxide, and amikacin, bedaquiline, beta-lactamase inhibitors such as avibactam, rifabutin, piperidinol-based compound-1, Indole-2-carboxamides and thiacetazone derivatives [10]. These drugs offer the choice and potential for new drug combinations to treat NTM diseases. Recently, many studies are initiated to understand the drug synergy of existing and new drugs at the *in vitro* (broth culture), inside macrophages, and in the zebrafish model. The information about new drugs, their PK/PD data, and validation in various models have been reviewed recently by Wu ML *et al*., which would be useful for any new study on NTM drug treatment [10]. Another avenue that has the potential to improve the clinical outcome of NTM therapy and/or to reduce the treatment duration is HDT, which was earlier discussed elaborately for TB [124, 127, 128]. Studies focussing on the repurposing of drugs that can modulate the host response should also help to improve the management of NTM diseases.

As much as rapid diagnosis and better treatment of an infectious disease are crucial, vaccines are very critical to contain the spread of infection and, thus, gain global health priority. BCG, a live attenuated strain of *M. bovis*, is the only vaccine approved by the WHO to prevent TB, mainly in children. At present, BCG is the most widely administered vaccine, with 90% coverage of the world population [138]. Although BCG is suitable for uninfected people, it cannot provide lifelong immunity and is unsuitable for HIV-infected patients. The development of newer vaccines aims at either a new form of recombinant BCG (rBCG) or ones that boosts the conventional BCG vaccine. Several potential rBCG/BCG vaccine candidates with novel adjuvants are currently being evaluated for use in TB prevention in clinical studies [139–142]. As previously stated, studies using mouse models have indicated the cross-reactive immunity offered by BCG to pulmonary NTM infections caused by *M. avium*, *M. abscessus,* and *M. kansasii* [82, 143]*.* Recently, a subunit vaccine (ID91) with the Toll-like receptor agonist glucopyranosyl lipid adjuvant formulated as ID91+GLA-SE indicated protection against *M. avium* in a mouse model [144]. However, unlike TB, the vaccine development pipeline is very poor for NTM infections, concerning the number and complexity of candidate vaccines. Thus, more research focus is needed in this area.

## Conclusion

Recently, the World Health Organization (WHO) has announced an “END-TB strategy” with targets to reduce TB deaths by 95% and to reduce new cases by 90% between 2015 and 2035 [73, 145]. However, the emergence of MDR and XDR TB, as well as co-existence of TB with other chronic diseases, pose a significant challenge in achieving this goal. Recent clinical findings have demonstrated the involvement of several NTM species in causing pulmonary diseases, which further contributes to the morbidity and mortality. Since NTMs are also known to have smear positivity and lung involvement very similar to Mtb*,* diagnosis of NTM in the clinical specimen is often misleading and underestimates the incidence of NTM in many countries. However, the global TB management strategy, including case finding/report, diagnosis, and treatment, are more advanced and better equipped than that available for NTM infections. The currently available tools for the diagnosis, species identification, and DST of NTM are under-developed. Besides, patients with NTM disease are treated with anti-TB drugs, although NTM does not respond well to the standard anti-TB drugs [10].

Nonetheless, a spike in research and clinical activities is noted recently for NTM infections, including the evaluation of a new class of drugs and the exploration of HDT as an alternative or adjunct to antibiotic therapy. These efforts are expected to improve the management of NTM infections globally in the future.

## Data Availability

Not applicable

## Abbreviations

TB: Tuberculosis
Mtb: Mycobacterium tuberculosis
NTM: Non-tuberculous mycobacteria
HIV: Human immunodeficiency virus
LTBI: Latent Mtb infection
WHO: World health organization
COPD: Chronic obstructive pulmonary disease
CF: Cystic fibrosis
DM: Diabetes mellitus
INH: Isoniazid
RIF: Rifampicin
PZA: Pyrazinamide
ETH: Ethambutol
MDR: Multidrug-resistant
XDR: Extremely drug-resistant
RR: Rifampicin resistant
PNB: P-nitro benzoic acid
ATS: American thoracic society
IDSA: Infectious diseases society of america
AFB: Acid-fast bacilli
FDA: Food and drug administration
NAAT: Nucleic acid amplification test
MTD: Mycobacterium tuberculosis direct
LAMP: Loop-mediated isothermal amplification
CPA: Cross-priming amplification
LiPA: Line probe assay
WGS: Whole genome sequencing
NGS: Next-generation sequencing
MALDI-TOF: Matrix-assisted laser desorption ionization-time of flight
MS: Mass spectrometry
TST: Tuberculin skin test
IGRA: Interferon-gamma release assay
PPD: Purified protein derivative
DST: Drug susceptibility testing
MODS: Microscopic observation of drug susceptibility
HDT: Host directed therapy
HRCT: High resolution computer tomography
